# Assessing decision regret in caregivers of deceased German people with cancer—A psychometric validation of the Decision Regret Scale for Caregivers

**DOI:** 10.1111/hex.12941

**Published:** 2019-07-31

**Authors:** Markus W. Haun, Alexander Schakowski, Ariane Preibsch, Hans‐Christoph Friederich, Mechthild Hartmann

**Affiliations:** ^1^ Department of General Internal Medicine and Psychosomatics Heidelberg University Heidelberg Germany

**Keywords:** aggressiveness of care, cancer, caregiver, confirmatory factor analysis, decision regret, measurement invariance

## Abstract

**Background:**

Decisional regret during or after medical treatments is linked to significant distress. Regret affects not only patients but also caregivers having an active or passive role during decision making. The Decision Regret Scale (DRS) is a self‐report measure for regret in patients after treatment decisions. However, practical and psychometrically robust instruments assessing regret in caregivers are lacking.

**Objective:**

To develop and validate a caregiver version of the DRS (Decision Regret Scale for Caregivers [DRS‐C]).

**Design:**

Psychometric validation based on a web survey.

**Setting and participants:**

361 caregivers of deceased German people/patients with cancer.

**Main variables studied:**

Besides structural validity and test‐retest reliability, we evaluated measurement invariance accounting for gender, age and closeness of relationship, and tested hypotheses on convergent/discriminant validity.

**Results:**

Forty‐five per cent of all caregivers demonstrated decision regret. Confirmatory factor analyses strongly supported the unidimensional structure of the DRS‐C and pointed to strict invariance. The DRS‐C demonstrated very good internal consistency (*α* = 0.83, 95% CI [0.81, 0.86]) and test‐retest reliability (ICC [A,1] = 0.73, 95% CI [0.59, 0.83]) along with sound convergent/discriminant validity. Concerning responsiveness, DRS‐C scores remained stable over a 12‐week period in 83.3% of all caregivers. Receiver operating characteristic analysis yielded a cut point of 43 for the identification of significant decision regret (AUC = 0.62, 95% CI [0.56, 0.68]).

**Discussion and conclusions:**

The lack of a gold standard instrument prevented us from examining the criterion validity and determining a minimally important difference. Nevertheless, the DRS‐C provides valid and reliable information regarding caregiver regret following medical decisions. Above all, it captures a crucial aspect of the treatment experience in caregivers.

## 1 INTRODUCTION

Many patients with serious illnesses (eg cancer diseases) and their caregivers are regularly confronted with difficult treatment decisions particularly when it comes to end of life. In the light of an unfavourable outcome, decision‐related regret becomes more likely. Connolly and Reb have defined regret as (a) aversive and avoided if possible, (b) an intimate interplay of thought and feeling, (c) distinct from other specific emotions, such as disappointment, and from general negative affect, and (d) based on a comparison of some event or process with another, better event or a process that ‘might have been’.^1^ Such comparisons occur regularly in the context of non‐hypothetical treatment decisions, for example in cancer care.^2^

For patients, the Decision Regret Scale (DRS) has been found to be a valid and reliable measure with a unidimensional structure and very good internal consistency.^3^, ^4^ Findings on associations between the DRS and comparator instruments point to sound construct validity. The DRS has been translated into seven languages and adapted for the application in various cultural contexts.^2^ The DRS has been applied in observational studies, for example, demonstrating that higher physician empathy predicts lower decision regret in people with cancer or patients with cancer.^5^, ^6^ The DRS has also been used in randomized controlled trials of decision aids which recently have been shown to reduce decisional conflict.^7^, ^8^, ^9^ Indeed, decision regret often reaches high levels (increasing 6 months or more after a decision) due to very poor outcomes and definitive knowledge about the outcome.^10^ Several risk factors have been identified as temporal predictors for decisional regret including decisional conflict, unmet information needs, serious adverse physical health outcomes and anxiety.^11^ Notably, decision regret is commonly not associated with patients’ sociodemographic characteristics but regularly linked to decreased mental well‐being making.^11^

Along with patients, caregivers are often affected by decision regret in the course or in the aftermath of participating in treatment decisions.^12^, ^13^ Indeed, one study demonstrated that caregivers experiencing decision regret have a lower health‐related quality of life.^14^ Furthermore, there is some recent evidence that decisional conflict in family members is negatively linked to their quality of end of life communication.^15^ However, research on decision regret in caregivers is scarce since an equally practical and psychometrically robust instrument for its assessment is lacking.^4^, ^11^, ^16^ Although one scale has been proposed some years ago, it was validated in a Japanese population only.^14^ Given the cultural differences, the generalizability of this instrument to Western countries is questionable. Overall, the few available measures for the emerging field of decision regret research in caregivers seem not to have been evaluated sufficiently.

To fill this gap, this study aimed to derive a caregiver‐adapted version of the widely used DRS originally conceptualized for patients.^2^, ^3^ Based on a comprehensive psychometric validation, we propose the robust and easy‐to‐administer Decision Regret Scale for Caregivers (DRS‐C) as a new instrument for measuring decision regret in caregivers.

## 2 METHODS

### 2.1 Research design and recruitment

#### 2.1.1 Study design

In a cross‐sectional web survey, we examined the psychometric properties of the DRS‐C. Besides assessing the structural validity in a confirmatory factor analysis (applying the factorial structure of the original DRS) and examining test‐retest reliability, we also conducted a cut point based on a receiver operating characteristic analysis. For evaluating construct validity and responsiveness over time, we pre‐defined hypotheses specifying directions and magnitude with comparator instruments.

#### 2.1.2 Sample size estimation

Accounting for confirmatory factor analysis, we followed established conventional criteria and determined 300 individuals as required minimum sample size.^17^ Assuming a type 1 error of 5% (two‐tailed) with a power of 0.80 and a total sample size of 300 observations, we were able to detect an effect size of *r* = 0.16 for Pearson product‐moment correlation coefficients.

#### 2.1.3 Sampling procedure

We collected data from 3 June 3 until 10 December 2015 and, for the retest, from 26 August 2015 until 3 March 2016. The median time interval between test and retest was 12 weeks. The sampling procedure followed a non‐probability approach where individuals were recruited through volunteering in an online survey provided via the web‐based interface SurveyMonkey^®^. Specifically, we identified 21 German grief support websites and online groups in a systematic online search and then invited caregivers of deceased individuals through postings on these websites (Appendix S1). The web survey implementation followed the guidelines proposed by Dillman et al (2014).^18^ To ensure unbiased answers and prevent measurement error, we did not offer any incentives to increase the attractiveness of participation.

#### 2.1.4 Reporting and ethical standards

We report the psychometric assessment in accordance with the Consensus‐based Standards for the selection of health status Measurement INstruments, COSMIN.^19^ The study was approved by the Institutional Review Board of the Medical Faculty of Heidelberg University, Heidelberg, Germany. All participants had the opportunity to ask questions prior to enrolment and gave their informed consent prior to assessment. They could withdraw from the study at any time.

### 2.2 Participants

The approached sample amounted to 559 eligible individuals with a subsequent completion rate of 65.3% (N = 365). After removal of four multivariate outliers, the data set for statistical analysis comprised 361 individuals. Participants were adults, aged 18‐79 (M = 46.7, SD = 10.9), who were caregivers of deceased German people with cancer or patients with cancer aged 18 years or older at the time of diagnosis. We applied the caregiver definition of the American Cancer Society.^20^ We did not enrol any living people with cancer or living patients with cancer. Exclusion criteria comprised a time period of <6 months between the patient's death and the enrolment of the bereaved. Table 1 describes participant characteristics for the sample.

**Table 1:** Participant characteristics for the sample (n = 361)

Characteristics	N	%	M	SD	Median	kewness
Age in years			46.7	10.9	8.0	−0.26
Gender						
Female	326	90.3				
Male	35	9.7				
Relationship status						
Married/de facto/living together	214	59.3				
Single	139	38.5				
Employment status						
Full time/self‐employed/student/domestic work	296	82.0				
Unemployed	14	3.9				
Pensioner	28	7.8				
Other	23	6.4				
I am the … of the deceased.						
Spouse/partner	133	36.8				
Daughter	115	31.9				
Mother or father	28	7.8				
Sibling	26	7.2				
Son or stepson	17	4.7				
Grandchild	14	3.9				
Friend	8	2.2				
Sister‐ or brother‐in‐law	6	1.7				
Son‐ or daughter‐in‐law	4	1.1				
Niece or nephew	4	1.1				
Godson or goddaughter	2	0.6				
Spouse of godson	1	0.3				
Not specified	1	0.3				
Age of deceased in years			59.5	4.3	60.0	−0.21
Time between initial tumour diagnosis and death of deceased in months			33.5	42.7	18.0	2.8
Time between death of deceased and survey participation in months			43.6	48.4	27.0	2.8
Tumour type						
Lung and bronchus	68	18.8				
Pancreas	38	10.5				
Brain	30	8.3				
Breast	27	7.5				
Rectum	23	6.4				
Haematological malignancy	20	5.5				
Stomach	19	5.3				
Liver	17	4.7				
Gynaecologic other than breast	16	4.4				
Ear Nose Throat (ENT)	13	3.6				
Prostate	12	3.3				
Soft tissue malignancy	12	3.3				
Other	66	18.3				
DRS**‐**C total score	361		9.5	6.1	40	0.32
DRS**‐**C total score ≥ 43	163	45.2				

Note

M and SD are used to represent mean and standard deviation, respectively.

Abbreviation: DRS**‐**C, Decision Regret Scale for Caregivers.

### 2.3 Measures

#### 2.3.1 Decision Regret Scale for Caregivers (German version)

The DRS‐C is the caregiver version of the Decision Regret Scale originally developed for patients by Brehaut and colleagues (the DRS‐C is available in Appendix S2).^3^ The DRS‐C is a unidimensional, self‐report instrument consisting of five items, which are answered on a 5‐point bipolar intensity scale. Completers evaluate the item statements by circling a number from 1 *(strongly agree*) to 5 (*strongly disagree*). Items 2 and 4 are phrased in the negative direction to avoid acquiescence bias. After reversing the scores of these two items, the overall sum score is produced by taking the mean of the five items and converting it to a score ranging from 0 to 100 by subtracting 1 and multiplying by 25. For the original DRS, estimates of internal consistency are within good limits (ie Cronbach's α ≥ 0.80), while the stability of the scale over time, that is test‐retest reliability, was not measured in the original validation. Construct validity has been assessed through correlation with the Satisfaction with Decision Scale (*r* = −0.67‐*r *= −0.40) and the Decisional Conflict Scale (*r *= 0.31‐*r *= 0.52). Criterion validity with overall quality of life has shown an association ranging from *r *= −0.30 to *r *= −0.25.

#### 2.3.2 Satisfaction with decision scale (German version)

The Satisfaction with decision scale (SWD) measures satisfaction with health‐care decisions independent from a good or bad decision outcome.^21^ This unidimensional, self‐report instrument includes six items, which are rated on a 5‐point bipolar intensity scale. In the original validation study, internal consistency as measured in Cronbach's alpha was at *α* = 0.88 indicating good reliability. Construct validity has been assessed through correlation with the Confidence in Decision Scale (*r *= 0.64) and Decisional Conflict Scale (*r *= −0.54). In our study, we removed the fourth item on the expected success in carrying out decisions from the SWD since we inquired about decision making in the past.

#### 2.3.3 Inventory of complicated grief (German version)

The ICG‐D is a self‐report questionnaire that can be used to screen patients for complicated grief.^22^, ^23^ This unidimensional instrument includes 19 items. Individuals describe the currently experienced emotional, cognitive and behavioural states on a 5‐point unipolar frequency scale. In the original validation study, internal consistency indicated excellent reliability (Cronbach's *α* = 0.94). Convergent validity has been assessed through correlation with the Beck Depression Inventory (*r *= 0.67), the Texas Revised Inventory of Grief (*r *= 0.87), the Grief Measurement Scale (*r *= 0.70) and, for the German version, the Global Severity Index of the Symptom Checklist‐90‐R (SCL‐90‐R; *r = *0.37).

#### 2.3.4 Patient health questionnaire‐9 (German version)

The PHQ‐9 is a widely used brief depression severity measure with high validity and reliability which scores each of the nine DSM‐IV criteria as ‘0’ (not at all) to ‘3’ (nearly every day).^24^

#### 2.3.5 Generalized anxiety disorder‐7 (German version)

The widely applied 7‐item GAD‐7 is a practical and valid self‐report anxiety questionnaire with unidimensional structure and good internal consistency (*α* = 0.89).^25^, ^26^ On a 4‐point unipolar intensity, individuals indicate how often they have experienced symptoms of generalized anxiety during the last 2 weeks.

### 2.4 Data analysis

The data preparation included screening for normality and outliers. First, we followed recommendations to inspect univariate distributions.^27^ Specifically, we assumed multivariate normality if skewness and kurtosis for the DRS‐C item values fell in the normal range (−2 to 2 and −7 to 7, respectively).^28^ Additionally, we computed Mahalanobis *D* ^2^ and detected four multivariate outliers among the 365 respondents. These outliers were deleted prior to the subsequent analyses. Second, to account for potential missing data due to item non‐response and gain efficiency relative to complete‐subject analysis, we applied full information maximum‐likelihood (FIML) estimation for incomplete data as part of the confirmatory factor analyses. FIML treats observations with random missing values. For descriptive statistics, we summarized results for discrete variables in absolute and relative frequencies, while for continuous variables, we provided means, standard deviations, medians and interquartile ranges.

**Figure 1:**
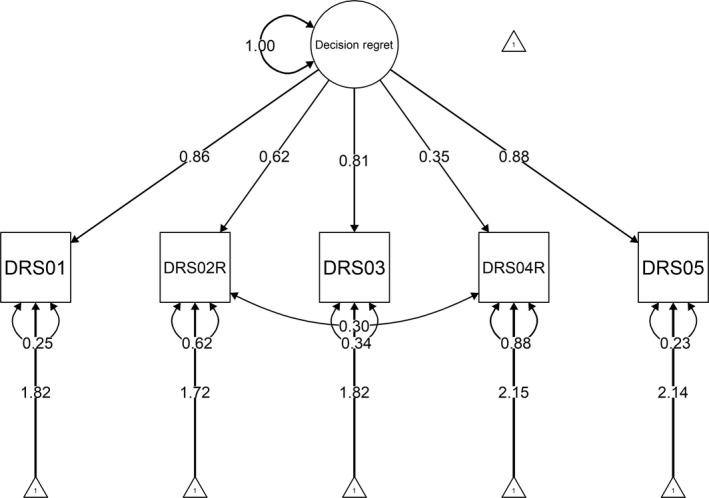
Final confirmatory factor analysis model for the Decision Regret Scale for Caregivers (DRS‐C) with path coefficients presented in standardized form

The psychometric validation of the DRS‐C followed classical test theory. With respect to construct validity, based on previous theoretical and psychometric work on the original DRS, we assumed that all items (interval scale) of the DRS‐C together comprehensively reflected decision regret as latent construct (interval scale). To investigate structural validity of the DRS‐C, we analysed covariance structure in a CFA and measurement invariance in multi‐group CFAs using the R packages *lavaan, semPlot and semTools*.^29^, ^30^, ^31^ Specifically, we tested the simple and plausible unidimensional model of the original DRS in which the five items as observed variables identified the latent factor of decision regret (see Figure 1). For FIML model evaluation, we calculated regression coefficients/loading estimates along with common fit indices (chi‐square value, comparative fit index, CFI, for incremental fit, standardized root‐mean‐square residual, SRMR, for absolute fit and root‐mean‐square error of approximation, RMSEA, as residual‐based measure). Along with the variance captured by the factor structure,^32^ we calculated internal consistency reflected in Cronbach's *α* providing that unidimensionality had been identified through CFA. For reliability and measurement error, we assumed that regret scores were stable in the interim period. Hence, we computed the standard error of measurement along with the intraclass correlation coefficient ICC (A,1; two‐way mixed effects model with absolute agreement specified) for test‐retest reliability using the *irr* package.^33^, ^34^ We assessed DRS‐C scores on an independent second administration for 60 participants who had agreed to be followed up by the study team. Test conditions were similar compared with the initial assessment (same type of administration and web environment with the same instructions).

To assess convergent validity, we tested the following a priori specified hypotheses in bivariate Pearson product‐moment correlations. For magnitudes, we applied the thresholds for effect sizes introduced by Cohen.^35^ We postulated that the DRS‐C would correlate.

- Negatively with the SWD score. This association would amount to a large effect size as the SWD measures satisfaction with decisions.
- Positively with the ICG‐D score. This association would amount to a small‐to‐moderate effect size, since complicated grief and regret are somewhat related constructs. However, we expected a moderate effect size at the most, since complicated grief has some overlap with depression and is highly variable over time.^36^
- Positively both with PHQ‐9 and with GAD‐7. This association would only amount to a small effect size, since depression and anxiety exhibit a higher variability within‐subject over time compared with regret and both constructs are related but not identical with regret.

Regarding differences between groups, we additionally hypothesized that DRS‐C scores would be significantly higher in those bereaved caregivers who had witnessed aggressiveness of care (AOC) at the end of life of the deceased compared with those caregivers who had not. For measuring AOC, we applied established claim‐based indicators.^37^ We assessed between‐group differences in a univariate analysis of covariance (ANCOVA), adjusting for the effects of gender, age, tumour type and place of death of the deceased.

To assess responsiveness over time, we evaluated the hypothesis that DRS and SWD change scores from T1 to T2 would be significantly negatively correlated. To determine an optimal cut point, we used the *pROC* package for a receiver operating characteristic (ROC) analysis.^38^ ROC curves graphically depict a test's ability to correctly identify ‘true‐positive’ and ‘true‐negative’ individuals for various test cut points.^39^ We estimated the area under an ROC (AUC) as an indicator for the overall accuracy of the DRS‐C in predicting clinically significant decision regret that would require professional attention. Unfortunately, we were not able to determine a minimally important difference (MID) since no external clinical or patient‐based indicator that would have demonstrated MID in the target patient population existed as a potential anchor.

The statistical analysis of the data was conducted by two analysts independently (MWH and AS) using R, version 3.5.2.^40^ For all analyses, statistical significance was evaluated at a type 1 error of 5% (two‐tailed).

## 3 RESULTS

### 3.1 Content validity

The DRS‐C aims to measure decisional regret in caregivers of patients with any type of disease. The concept of decision regret covers the ‘negative emotion involving distress or remorse following a decision’ that is linked to dissatisfaction with medical decision making, lower quality of life and poorer health.^2^ Since we wanted to apply our instrument to a German population, we used a parallel translation approach to derive a German version of the DRS, which was then minimally adapted to derive the DRS‐C. 41 Allowing for the evaluation of a whole treatment period, we adapted the item wording slightly in order to inquire about multiple decisions rather than one. The original developer of the DRS, Jamie Brehaut, PhD, conducted a final check. For detailed information on the procedures to maximize cross‐national equivalence, see Appendix S3.

To maximize content validity of the DRS‐C, we relied on structured questionnaire pre‐testing which included cognitive interviewing to evaluate the instruction, the body of the individual items and the response format for recording the answers. Specifically, we assessed the relevance, representativeness/comprehensiveness and comprehensibility of the DRS‐C independently in two adults (woman aged 56 years with education >9 years; man aged 70 with education <9 years) who were medical laypersons and had recently experienced the death of a relative. We observed respondent behaviour and followed an active interviewing style with probes based on the content of the interview, that is initial participant responses. Please find more information on the pre‐testing in Appendix S4 and the protocol for the cognitive interviews including the suggested scripted probes in Appendix S5. In sum, participants answered in a straightforward manner without rejecting the premise of any questions. While we observed minor imprecision in some responses, participants generally had no major trouble with providing quantitative responses.

In our survey sample, we did not find any floor or ceiling effects indicating that no extreme items were missing in both ends of the scale. Descriptive statistics for the DRS‐C items are given in Table 2 and Appendix S6.

**Table 2:** Descriptive statistics for the DRS**‐**C items

Item No.	Item	*M*	SD	Range	Skewness	Kurtosis
1	The decisions were right	2.3	1.3	1.0‐5.0	0.71	2.45
2	I regret the choices that were made	2.3	1.3	1.0‐5.0	0.63	2.17
3	I would go for the same choices if I had to do it over again	2.7	1.5	1.0‐5.0	0.29	1.70
4	The choices did me a lot of harm.	3.0	1.4	1.0‐5.0	‐0.09	1.79
5	The decisions were wise ones	2.7	1.2	1.0‐5.0	0.26	2.19

Note

M and SD are used to represent mean and standard deviation, respectively.

Abbreviation: DRS**‐**C, Decision Regret Scale for Caregivers.

### 3.2 Structural validity

Following a reflective model, in which all items are a manifestation of the same underlying construct, we fitted the original theoretical model of the DRS to our data in order to evaluate whether the one‐factor structure also applied to the DRS‐C. Specifically, we hypothesized that the five items of the DRS‐C as the observed variables loaded on a single latent factor. Given the five variables, there were $\frac{5\times (5+1)}{2}=15$ data points while 10 parameters (four regression coefficients/factor loadings and six variances) were to be estimated. Hence, the model was overidentified and tested with 5 *df*. The ratio of cases to observed variables was 72:1 (36:1 for cases to estimated parameters). Both skewness and kurtosis indices did not indicate any violations from normality. Thus, we did not transform the data. In randomly selected pairs of scatterplots, all observed variables appeared to be linearly related. Pearson product‐moment correlations did not reveal evidence for collinearity/singularity. In the CFA, the independence model that tested the hypothesis that all variables are uncorrelated was rejected, *χ* ^2^ (10, N = 361) = 855.20, *P *< .0001. We then tested the hypothesized model. However, fit measures indicated some misspecification, *χ* ^2^ (5, N = 361) = 39.42, *P *< .0001, CFI = 0.96, SRMR = 0.05, RMSEA = 0.14, 90% CI (0.10, 0.18). Residual diagnostics traced this misspecification to the relationship between the indicator residual variances for items 2 and 4. We assumed non‐random measurement error due to their reverse‐wording, which is a common observation.^42^ Hence, in our only respecification, we specified error covariances between items 2 and 4, which led to a significantly improved fit, *χ* ^2^ (4, N = 361) = 8.56, *P *= .073, CFI = 0.99, SRMR = 0.01, RMSEA = 0.056, 90% CI (0.000, 0.109; the sample covariance matrix is available in Appendix S7). The chi‐square difference test indicated a significant improvement in fit between the hypothesized model and the model including the error covariance, Δ*χ* ^2^ (1, N = 361) = 30.86, *P *< .0001. A graphic representation of the final measurement model including standardized factor loadings for the observed variables can be found in Figure 1. In line with the combination rule of a CFI > 0.95 and a SRMR < 0.08 suggested by Hu and Bentler,^43^ the model demonstrated a good fit with standardized factor loadings ranging from 0.35 to 0.88. Four out of five loadings were well above 0.50 (conventionally indicating a strong relation between indicator and construct), and all were statistically significant (*z‐*values from 6.5 to 45.1, all *P *< .0001). The single factor captured 53.5% of the variance in relation to measurement error variance.

### 3.3 Internal consistency

Internal consistency of the DRS‐C scale as measured by Cronbach's alpha amounted to *α* = 0.83, 95% CI (0.81, 0.86), which can be considered very good.^44^

### 3.4 Cross‐cultural validity/measurement invariance

To evaluate across‐group equivalence of the parameters, we tested for measurement invariance employing multi‐group confirmatory factor analyses across gender (male vs. female), age groups (under 50 years old vs. 50 years or older) and closeness of relationship (spouse/first‐degree relative vs. other; Appendix S8).^45^, ^46^ In sum, we concluded that the respondents from different groups with same the latent level of decision regret responded similarly to a particular item of the DRS‐C. Since we found strict invariance, we then tested for the equality of latent means and did not find any significant differences between the groups (two‐sample *t* test for gender: *t*(359) = 0.29, *P *= .775, *d* = 0.03; age group: *t*(359) = −1.16, *P *= .247, *d *= −0.12; closeness of relationship: *t*(359)=0.13, *P *= .895, *d* = 0.01).

### 3.5 Reliability

The median time interval between test and retest was 12 weeks (interquartile range: 6 weeks), which we considered long enough to prevent recall bias, yet short enough to ensure that no change in regret had set in. With respect to test‐retest reliability, we identified robust measurement reproducibility for the DRS‐C as expressed in an ICC(A,1) of 0.71 (N = 60; two‐way random effects model; 95% CI [0.56, 0.82]).

### 3.6 Measurement error

The smallest detectable change in the DRS‐C score, defined as the change score beyond measurement error, was derived from the standard error of measurement of 10.74 (N = 60) and amounted to 29.76 for individuals and 5.29 for comparisons of mean scores between groups, respectively.

### 3.7 Criterion validity: Optimal cut point and receiver operating characteristic analysis

**Figure 2:**
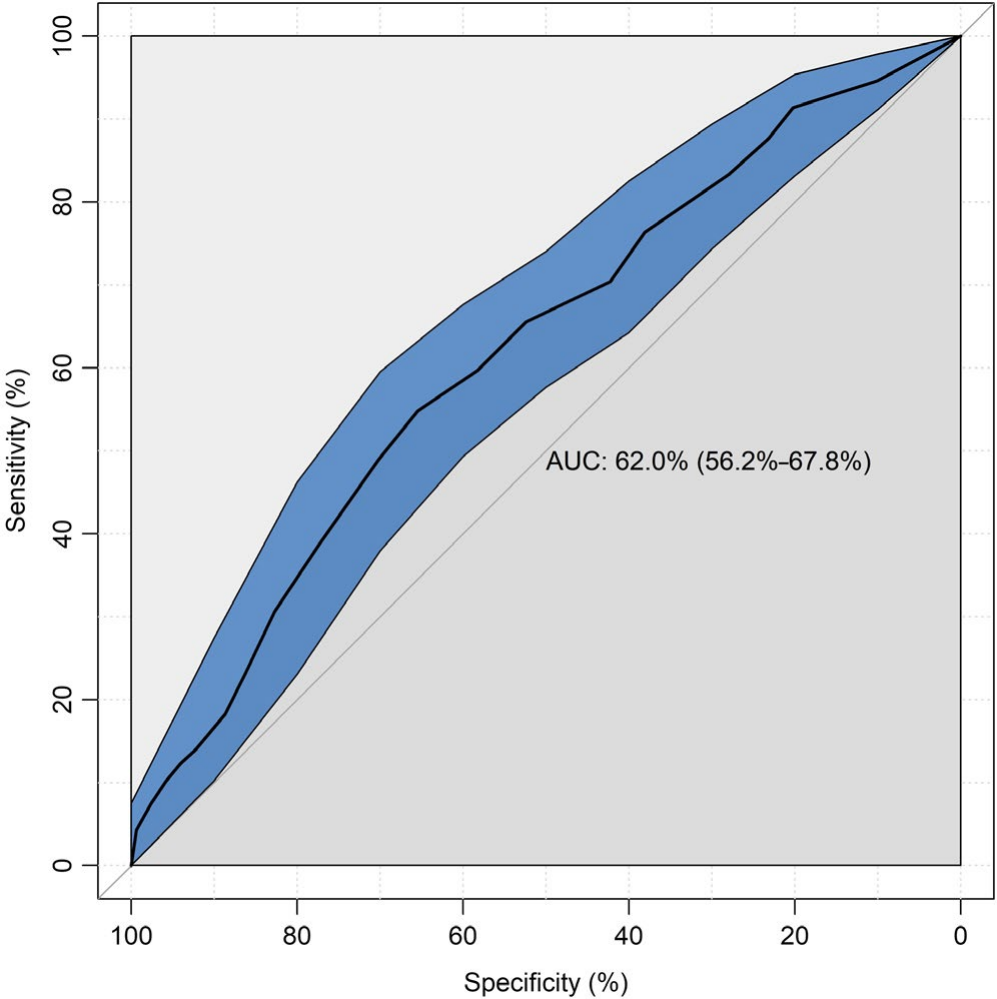
Receiver operator characteristic curve (ROC) for the Decision Regret Scale for Caregivers (DRS‐C). AUC = area under the curve

We conducted a ROC analysis to identify an optimal cut point on the DRS‐C that indicated clinically significant decision regret based on concomitant complicated grief (as indicated by an ICG‐D score > 25). The AUC for the DRS‐C was 0.62, 95% CI 95% [0.56, 0.68], indicating that the DRS‐C cut point was able to accurately discriminate individuals with clinically significant regret above random chance (see Figure 2 for the ROC curve). Applying the Youden Index as a measure of overall diagnostic effectiveness that gives equal weight to sensitivity and specificity, a score of 43 on the DRS‐C was linked with a positive predictive value of 0.64 and negative predictive value of 0.57 concerning clinically significant decision regret. Hence, a score of 43 can be applied as a cut point for screening and research purposes. In our sample, 45.2% of all participants exceeded this cut point.

### 3.8 Hypotheses testing for construct validity

Bivariate associations between the DRS‐C score and the comparator instruments can be found in Table 3. Since missing data per association did not exceed 3%, we performed pairwise‐complete correlations at this point. The findings supported three of our four hypotheses. However, for AOC, we did not find any differences between DRS‐C scores for those caregivers who had witnessed AOC compared with those who had not, F(4, *353*)=0.91, *P *= .46 (adjusted for gender, age, tumour type and place of death of the deceased).

**Table 3:** Means, standard deviations and correlations with confidence intervals

Variables	M	SD	1	2	3	4
1. DRS‐C Score	12.93	5.21				
2. ICG‐D Score	27.84	13.76	0.20**			
			[0.10, 0.30]			
3. GAD‐7 Score	7.39	5.39	0.17**	0.60**		
			[0.07, 0.27]	[0.53, 0.66]		
4. PHQ‐9 Score	8.48	6.71	0.14*	0.67**	0.82**	
			[0.03, 0.24]	[0.61, 0.72]	[0.79, 0.86]	
5. SWD Score	10.86	6.27	‐0.64**	‐0.13*	‐0.12*	‐0.09
			[−0.69, −0.57]	[−0.23, −0.02]	[−0.22, −0.01]	[−0.19, 0.02]

Note

M and SD are used to represent mean and standard deviation, respectively. Values in square brackets indicate the 95% confidence interval for each correlation.

Abbreviations: DRS‐C, Decision Regret Scale for Caregivers; GAD‐7, Generalized Anxiety Disorder‐7; ICG, Inventory of Complicated Grief; PHQ‐9, Patient Health Questionnaire‐9; SWD, Satisfaction with Decision Scale.

* Indicates *P* < .05.

** Indicates *P* < .01.

### 3.9 Responsiveness

For the 60 participants for whom two measurements were available, we considered changes over time in DRS‐C scores as significant if they exceeded the measurement of 29.76 points. By this standard, 83.3% (N = 50) of all participants remained stable, while 10% (N = 6) improved and 6.7% (N = 4) deteriorated. We found support for our hypothesis with a significant negative correlation of *r *= −0.27, 95% CI [−0.49, −0.02], *P *= .035, between DRS and SWD change scores from T1 to T2. Additionally, we observed a positive correlation of *r *= 0.24, 95% CI [−0.01, 0.47], *P *= 0.063, between DRS and ICG‐D change scores from T1 to T2. The Bland‐Altman analysis yielded a mean difference DRS‐C score between T1 and T2 $\overset{¯}{d}$ of 0.92 points, 95% CI [−40.09; 41.92], indicating no systematic bias between the two administrations of the questionnaires (reflected in the 95% CI containing the zero). To the best of our knowledge, there was no intervention or other systematic exposure in the interim period between the two measurements. All comparator instruments were robust, that is psychometrically validated measures with balanced scales that prevent artificially extreme ratings.

## 4 DISCUSSION

To address the need for a valid and reliable self‐report measure of decision regret in caregivers, the current study assessed the psychometric properties of the DRS‐C, an adapted version of the original DRS developed for patients.

With respect to structural validity, the CFA confirmed a unidimensional structure of the DRS‐C similar to the DRS. Prior to our work, unidimensionality of the original DRS was also confirmed in internal cardioverter defibrillator recipients and people with cancer or patients with cancer receiving adjuvant chemotherapy.^4^, ^47^ The internal consistency was very good and comparable to the one reported for the original DRS (0.81 to 0.84 for people with cancer or patients with cancer) and the one identified in the Japanese validation of the original DRS (0.85).^3^, ^48^ Reproducibility for the DRS‐C over an average 12‐week period was considerable, indicating reasonable stability over time which corroborates previous findings in a patient sample with localized breast cancer 49 and from a recent systematic review.^2^ However, at this point, it remains plausible that regret diminishes over time in many individuals as some authors have argued.^2^ Concerning construct validity, findings supported our a priori hypotheses on the direction and magnitude of associations between the DRS‐C and comparator instruments. Hence, our findings point to convergent and discriminant validity of the DRS‐C. Correlation coefficients between regret and comparator instruments were comparable to those detected in the original validation study for the DRS (eg −0.67 to −0.40 and −0.23 to −0.17 for the SWD and psychological health, respectively).^3^ However, we did not detect between‐group differences for bereaved caregivers who had witnessed AOC at the end of life of the deceased and those caregivers who had not. One possible explanation may be the median time of 28 months between the death of deceased and participation in our survey. It seems plausible that even those bereaved caregivers who had experienced aggressive care at the end of the life of their significant others and had experienced decision regret in the aftermath subsequently became more accepting of the treatment decisions related to aggressive care.

To the best of our knowledge, there is only one previous study on decision regret in bereaved family members that used the Decision Regret Scale.^15^ The study by Smith‐Howell et al reported a lower mean decision regret score (M = 22.2, SD = 17.8) compared with the one we observed. This difference might be due to the fact that this study recruited in palliative care where, in comparison with other medical specialties, caregiver involvement in decision making is much more common.^50^ For the most part, decisions in palliative care are based on the needs of patients *and* caregivers so that regret might generally be lower in this particular context.^51^ At any rate, Smith‐Howell et al did not report whether they had validated their measure in a caregiver sample in advance.^15^ This study was published after we had completed our data collection, so that we could not account for it when pre‐testing the DRS‐C. We found one study that explored decision regret in family members actively taking care of people with cancer or patients with cancer.^52^ For this small sample of head and neck cancer patient caregivers, mean regret scores were significantly lower (M = 10.5, SD = 9.9) compared with our sample (N = 30). However, patients were treated with curative intent, data were collected by clinicians, and the authors did not report whether they had validated their DRS version for caregivers. In a study of surrogate decision makers for the chronically critically ill, the mean decision regret one week after making a tracheostomy or feeding tube decision also was significantly lower (M = 16.3, SD = 11.6) compared with our sample.^53^ The content of the decision making was less comparable to the one under investigation in our study. Notably, the sample size was very small (N = 16), which is why we did not consider perceived effective decisions, which was identified as predictor for regret, for further refinement of the DRS‐C.

All in all, previous findings on decision regret either were developed in caregivers actively looking after people with cancer or patients with cancer or concern less existential scenarios. The somewhat higher regret scores in our sample may be due to caregivers having not been able to make an informed choice for their loved ones, since shared decision making is not necessarily implemented on a regular basis in German hospitals. In this regard, there is some evidence that shared decision making may potentially modify the decisional regret experience of bereaved family caregivers.^54^ Unfortunately, we did not assess whether an informed choice was made.

Some methodological limitations as sources of potential bias must be discussed. First, we did conduct only two cognitive interviews as part of pre‐testing by which we may have missed aspects of relevance and/or comprehensiveness. Nonetheless, we considered this acceptable as our instrument is very similar to the original DRS for which content validity has been investigated in previous work.^3^, ^4^ Second, our sampling followed a non‐probability approach as individuals volunteered in a web survey. Due to coverage error and potential self‐selection, sampling error may have occurred as collected data may stem from only a subset of the entire population and potentially lack representativeness. Specifically, non‐whites, people aged older than 65 years, people with lower incomes and those with less education are known to have lower Internet access rates than their counterparts and, therefore, are more likely to be under‐represented in web surveys.^55^ Indeed, our sample was somewhat younger compared with the ones in the original DRS validation studies.^3^ As in a comparable previous study,^14^ we observed an oversampling of women who were on average relatively young and tended to be full‐time working. However, DRS‐C scores in our sample were sufficiently dispersed and to date, for web surveys, there is no simple procedure available for drawing samples (comparable to random‐digit dialling) so that coverage/sampling error can be fully avoided.^18^ For the German population at least, to our best knowledge, there is currently no Internet panel allowing for probability‐based sampling. Furthermore, the population of bereaved caregivers itself cannot be well reached and drawing a sample from this population may be very difficult regardless of the sampling procedures. At any rate, our findings must be validated in a more comprehensive sample in future replications. Third, due to the nature of our sampling, we could not retrieve any information on non‐responding participants which impeded sensitivity analysis between participants and non‐respondents for the assessment of potential selection bias. Fourth, we relied on participants recollecting a remote exposure and hence cannot rule out information bias. Finally, the overall ability of the DRS‐C to discriminate between individuals with and without clinically significant decision regret was rather poor.^56^ At this point, we would therefore recommend the DRS‐C for research and screening purposes that are followed by a definitive assessment of clinical distress with other patient‐reported outcomes, only. The cut point we identified is somewhat higher than the ones that have been suggested previously.^2^ However, this can be explained by the rather common adverse outcomes in cancer treatment that contribute to higher decision regret. In fact, it has been recently proposed to apply cut points of 30 or higher.^2^

## 5 CONCLUSION

In sum, the findings of this study indicate that the DRS‐C provides valid and reliable information regarding regret in caregivers following medical decisions in patients. The items of the DRS‐C proved to be empirically identifiable, and logical operationalizations capturing the key idea of the latent decision regret construct. The lack of an existing gold standard instrument prevented the determination of an MID. Nevertheless, we demonstrated that the DRS‐C has sound psychometric properties including measurement invariance, appropriate responsiveness and interpretability. To the best of our knowledge, our study is also the first to determine a cut point for a DRS version based on a ROC analysis: although at this point, we only recommend the DRS‐C for research and screening on regret after (non)‐hypothetical decisions, the instrument is a psychometrically robust and easy‐to‐complete patient‐reported outcome. Above all, it captures an important aspect of the subjective treatment experience in caregivers.

## CONFLICT OF INTEREST

The authors declare that there is no conflict of interest.

## Data Availability

The data that support the findings of this study are available on request from the corresponding author. The data are not publicly available due to privacy or ethical restrictions.
