# Supplementary material for: Assessing decision regret in caregivers of deceased German people with cancer—A psychometric validation of the Decision Regret Scale for Caregivers
Source: Health Expect. 2019 Jul 31;22(5):1089–99. doi: 10.1111/hex.12941 (PMC6803409; doi:10.1111/hex.12941)
Supplement: Supplementary file 1 [file HEX-22-1089-s001.docx]

**APPENDICES**

***Appendix S1.* Overview of websites used for participant recruitment**

| No. | Website | Operated by | Coverage | Description |
| --- | --- | --- | --- | --- |
| 1 | https://forum.aspetos.com | commercial business | 8,566 members | message board for bereaved people |
| 2 | http://www.trauergruppe.de/ | private | n/a | nationwide register for grief-related encounter groups |
| 3 | https://www.trauer.de/ | commercial publisher | n/a | message board for bereaved people |
| 4 | http://www.infrieden.de/Trauer-und-Trost/Internetlinks | commercial publisher | n/a | webpage offering information on grief-related topics |
| 5 | https://chatseelsorge.evlka.de/v2/index.php | Protestant church | n/a | message board for bereaved people moderated by professional grief counselors |
| 6 | http://www.verwitwet.de/baseportal/home | non-commercial association | 11,217 members | web community for widows and widowers |
| 7 | http://allesistanders.de/ | non-commercial association | n/a | message board for younger bereaved people [active until 2018] |
| 8 | http://www.trauernetz.de/aktuell.html | Protestant Church | n/a | web page for caregivers and bereaved people, operated by the church |
| 9 | https://forum.verwitwet-info.de | private | 633 members | message board and message board for widowers and widows |
| 10 | http://bfriends.brigitte.de/foren/tod-trauer-trauerbewaeltigung/32211-anmerkungen-trauerforum.html | commercial publisher/magazine | n/a | web page and message board for bereaved people |
| 11 | http://www.gofeminin.de/forum/show1_f96_1/psychologie/verlust-einer-geliebten-person.html | commercial business | n/a | web forum containing information on psychology and related topics, grief-related message board |
| 12 | http://www.trauer.org/ | private | n/a | web page for bereaved people and message board moderated by grief-counselors [currently archived] |
| 13 | http://trauer.hna.de/Anzeige-suchen/Letzte-Ausgabe/1  http://trauer.hna.de/Board.aspx | commercial publisher/newspaper | n/a | message board for bereaved people moderated by grief counselors |
| 14 | http://www.leben-ohne-dich.de/foren.htm | non-commercial association | n/a | message board for bereaved children and brothers and sisters |
| 15 | https://www.hospiz-aktuell.de/herzlich-willkommen/?no_cache=1 | non-profit organization | n/a | web page containing information on grief-related topics |
| 16 | http://www.trauerbegleiter.org/inhalt/trauer_online | private | n/a | message board for bereaved people moderated by grief counselors |
| 17 | http://www.trauercafe.de/ | private | n/a | web page for bereaved people containing information on grief-related topics |
| 18 | http://www.trauerkreis.de/news.php | non-commercial association | 485 members | web page containing information on grief-related members and message board |
| 19 | https://www.zeit-trauer-raum.de/index.html | private | n/a | web page for bereaved people containing information on grief-related topics provided by grief counselors |
| 20 | http://wbb-foren.de/item/92-trauernetz-das-online-trauerforum-trauer-mit-teilen-mit-trauer-leben-trauer-begl/ | private | n/a | n/a [offline] |
| 21 | http://www.trauernetz.org/ | private | n/a | n/a [offline] |

*Note*. To find eligible websites, we conducted web searches with the following German keywords: trauerforum; trauerportal; online trauerportal; online trauerforum; online trauern; trauergruppe online; online seelsorge; online trauerkreis; trauerarbeit online; trauermagazin online.

***Appendix S2.* The Decision Regret Scale for Caregivers (DRS–C)**

| No. | Item | Strongly disagree |  |  |  | Strongly  agree |
| --- | --- | --- | --- | --- | --- | --- |
| 1 | The decisions were right. | 1 | 2 | 3 | 4 | 5 |
| 2 | I regret the choices that were made. | 1 | 2 | 3 | 4 | 5 |
| 3 | I would go for the same choice if I had to do it over again. | 1 | 2 | 3 | 4 | 5 |
| 4 | The choices did me a lot of harm. | 1 | 2 | 3 | 4 | 5 |
| 5 | The decisions were wise ones. | 1 | 2 | 3 | 4 | 5 |

***Appendix S3.* Detailed** **information on procedures to maximize cross-national equivalence**

To maximize functional cross-national equivalence, we first checked the compliance of the DRS-C items with the 12 guidelines for making items more translatable (e.g., “Avoid metaphors and colloquialisms”) introduced by Brislin (Brislin, R. W. (1986). The wording and translation of research instruments. In Lonner, W. J., & Berry, J. W. (Eds.), Field Methods in cross-cultural research (pp. 137-164). Beverly Hills, CA: Sage). Additionally, we ruled out any vague quantifiers, items with ambiguous/dual meanings, double-barreled questions, imprecise time references. Second, to focus on meaning rather than on an overly close translation and to emphasize optimal wording in the target language, we used a parallel translation approach with (1) translation of the DRS as source language questionnaire into German as target language by an English native speaker who is a freelance professional translator and also a seminar instructor on the topic of academic writing, (2) a second translation from English to German by a medical lay person, (3) comparison of the two source language questionnaires which included consulting the developers of original DRS, and (4) consistency checks and careful proofreading of the final translation. Both translators had received an initial briefing on the source questionnaire, its measurements components and the target audience.

For the bodies of the individual items, we did not encounter any major language ambiguities or incompatibilities. Specifically, we did not find (a) any syntactical or semantic ambiguity, (b) semantic polysemy (e.g., words with potentially equivalent literal meanings but different social implications, terms used to describe one object in Germany describing something else in English-speaking countries) or (c) differences in conditions and structure, i.e., the notion of regret and how one would ask about it potentially differing across countries. As for the response scales, the DRS-C – in line with the DRS – rates each term on a numerical scale with Likert-type question format (with 1 to 5 points) which is regarded as the best compromise “to increase the equivalency between responses to questions in cross-national research” (Smith, T. W. (2004). Developing and evaluating cross-national survey instruments. In Presser, S., Rothgeb, J. M., Couper, M. P., Lessler, J. T., Martin, E., Martin, J., & Singer, E. (Ed.), Methods for testing (pp. 89-108). Hoboken, NJ: John Wiley & Sons, Inc.). The DRS and DRS-C in their English versions apply “agree-disagree” scales, which can only be translated as “agree-not agree” in German, since German has no expression that formally matches “disagree”, only terms for “not agree” or “reject” are available. However, we did not expect major cross-national sources of measurement variation due to response effects. Specifically, we did not find any evidence of differences between Germany and other Western countries in social desirability, acquiescence bias, extreme response styles, “don’t know” responses/nonattitudes, neutral/middle options, question order, and mode of administration.

***Appendix S4.* Detailed information on the pretesting procedures**

First, we decided to observe respondent behavior with respect to contradictions and inconsistencies and to follow an active interviewing style selecting probes based on specific issues that arose in the interviews. The cognitive interviewing was done by the principal investigator who is a specialist in internal medicine and a certified psycho-oncologist, also holds a master’s degree in clinical and developmental psychology and is currently in the advanced stage of his training for board certification in psychosomatic medicine and psychotherapy. He has advanced knowledge in pretesting survey instruments and has authored two psychometric validations related to the assessment of unmet needs in caregivers of cancer patients and to communication in dyads affected by cancer. His level of education was typical of experienced practitioners of cognitive interviewing (Conrad, F. G., & Blair, J. (2004). Data quality in cognitive interviews: The case of verbal reports. In Presser, S., Rothgeb, J. M., Couper, M. P., Lessler, J. T., Martin, E., Martin, J., & Singer, E. (Ed.), Methods for testing (pp. 67-87). Hoboken, NJ: John Wiley & Sons, Inc.). At any rate, the background of the interviewer ensured close association with field which is regarded critical for performance in cognitive interviews (DeMaio, T. J., & Landreth, A. (2004). Do different cognitive interview techniques produce different results? In Presser, S., Rothgeb, J. M., Couper, M. P., Lessler, J. T., Martin, E., Martin, J., & Singer, E. (Ed.), Methods for testing (pp. 89-108). Hoboken, NJ: John Wiley & Sons, Inc.).

Second, in mock interviews as part of training, we ensured that the interviewer developed a thorough understanding of the instrument and potential design and response errors. We encouraged him to use his adaptive listening and investigate skills and instructed him to generally probe based on the content of the interview and initial participant responses. The interviewer was advised to use emergent unscripted probes about the meaning of the responses. Nevertheless, we also provided some scripted probes a priori. Please find the protocol for the cognitive interviews including the suggested scripted probes derived from a review of the original DRS in Appendix 5.

Third, during cognitive interviewing, the interviewer administered the probes immediately following questions applying the full range of follow-up probing techniques. For each interview, he provided written summaries.

Fourth, we did unfortunately not have the resources to record and behavior code the cognitive interviews for a true systematic analysis of interviewer and respondent behavior. However. we did conduct retrospective debriefings in which the interviewer related his experience with the questionnaire and offered his views about the questionnaire’s problems, that is, questions containing unwarranted suppositions, awkward wordings, or missing response categories. In addition, we reviewed the written summaries of the interviews for lexical (word meaning), logical, temporal and computational problems.

In sum, participants answered in a straightforward manner without rejecting the premise of any questions. While we observed minor imprecision in some responses, participants generally had no major trouble with providing quantitative responses. Indeed, no one failed to provide a codable response and the questions exhibited a considerable amount of analytic utility. Hence, we could maintain the question wordings proposed for the DRS-C.

***Appendix S5.* Protocol for the cognitive interviews**

**General remarks:**

- Accounting for the fact that information about retrieval processes is not available to respondents (e.g., Ericcson and Simon, 1993) and to limit your contribution to reactive effects, please intervene only when the respondents’ behavior indicates a potential problem leading to response error. Examples for indicators are:
  - an explicit statement of difficulty
  - indirect indications such prolonged silence, disfluent speech, high number of words uttered, doubt words, and switches in answer alternative
- If there are indications of a problem, please probe by describing the respondent behavior that suggested the possibility of a problem (e.g., “You took some time to answer, can you tell me why?”).
- To explore potential problems, please take an active role while interviewing by combining scripted and unscripted emerging probes in response to what the respondent says.
- Please focus on question flaws and pay extra attention if questions are problematic to subgroups of respondents with certain demographic or behavioral characteristics.
- Please note down the answers to the probes.

**Suggested scripted probes:**

- comprehension-oriented probes
  - “What were you thinking about while answering that?”
  - “How did you come up with your answer?”
  - “What does [term] mean to you?”
  - “Did you know the answer, or did you have to estimate?”
- confirmatory probes and mirroring what a participant said verbatim (repeating something the respondent said in a request for confirmation)
- reorienting probes (repetition or clarification of the task of providing an answer to the question within the response format provided)
  - “To what degree would you agree/disagree with the statement given the scale ranging from 1 to 5?“
- feedback to reinforce desirable response behavior
  - “Thanks that’s just the sort of information I am looking for”
  - “I know what you mean”
- Not until completion of the questionnaire (in order to judge whether participants could not or simply did not answer questions): elaborating probes
  - “Tell me more about that”

**After the cognitive interview**

- Please compile a written summary of your understanding of the verbal report provided by the participant. Specifically, list problems in each question of each interview.

***Appendix S6*. Histogram for the distribution of the Decision Regret Scale for Caregivers (DRS–C) score in the study population (dark red line indicates normal distribution)**


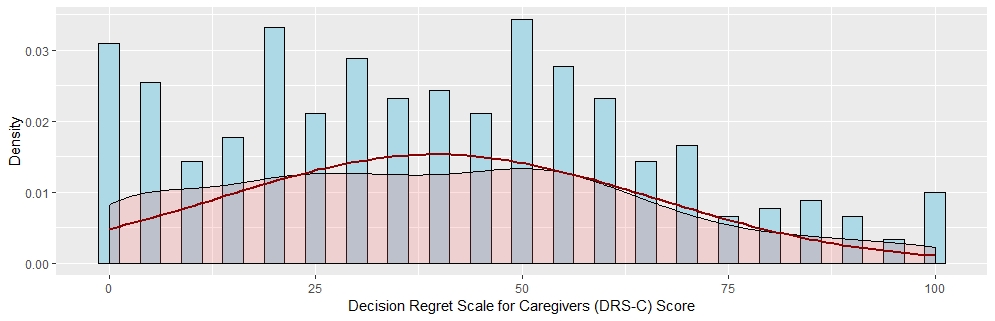


***Appendix S7.* Sample covariance matrix**

|  | DRS-C01 | DRS-C02 | DRS-C03 | DRS-C04 | DRS-C05 |
| --- | --- | --- | --- | --- | --- |
| DRS-C01 | 1.58 |  |  |  |  |
| DRS-C02 | 0.91 | 1.77 |  |  |  |
| DRS-C03 | 1.27 | 1.07 | 2.17 |  |  |
| DRS-C04 | 0.52 | 0.81 | 0.60 | 1.96 |  |
| DRS-C05 | 1.19 | 0.84 | 1.31 | 0.54 | 1.54 |

*Note:* DRS**–**C = Decision Regret Scale for Caregivers.

***Appendix S8*. Table for measurement invariance assessment (model comparisons in multigroup confirmatory factor analyses)**

| Gender: Model | χ²*(df)* | CFI | RMSEA  (90% CI) | SRMR | Model comp | Δχ²(Δ*df*) | ΔCFI | ΔRMSEA | Decision^#^ |
| --- | --- | --- | --- | --- | --- | --- | --- | --- | --- |
| M1 Configural invariance | 13.14(8) | .994 | .060  (.000-.115) | .02 | – | – | – |  | – |
| M2 Weak invariance (loadings) | 16.02(12) | .995 | .043  (.000-.093) | .03 | M1 | 2.87(4) | .001 | .017 | Accept |
| M3 Strong invariance (loadings  and intercepts) | 24.44(16) | .990 | .054  (.000-.095) | .03 | M2 | 8.42(4) | .005 | .011 | Accept |
| M4 Strict invariance (loadings, intercepts and residuals) | 36.91(21) | .981 | .065  (.027-.099) | .03 | M3 | 12.47(5)* | .009 | .011 | Accept |

*Note.* *N* = 361; group 1 (women) *n* = 326; group 2 (men) *n* = 35. * = *p* ≤ .05. *df* = degrees of freedom; CFI = Comparative Fit Index; RMSEA = Root Mean Score Error of Approximation; SRMR = Standardized Root Mean Residual. ^#^According to Cheung and Rensvold (2002) ΔCFI < 0.01 implies that the invariance assumption still holds.^1^

| Age group: Model | χ²*(df)* | CFI | RMSEA  (90% CI) | SRMR | Model comp | Δχ²(Δ*df*) | ΔCFI | ΔRMSEA | Decision^#^ |
| --- | --- | --- | --- | --- | --- | --- | --- | --- | --- |
| M1 Configural invariance | 14.65(8) | .992 | .068  (.000-.122) | .02 | – | – | – |  | – |
| M2 Weak invariance (loadings) | 20.00(12) | .991 | .061  (.000-.106) | .04 | M1 | 5.35(4) | .002 | .007 | Accept |
| M3 Strong invariance (loadings  and intercepts) | 21.31(16) | .994 | .043  (.000-.086) | .04 | M2 | 1.31(4) | .003 | .018 | Accept |
| M4 Strict invariance (loadings, intercepts and residuals) | 28.34(21) | .991 | .044  (.000-.082) |  | M3 | 7.04(5) | .002 | .001 | Accept |

*Note.* *N* = 361; group 1 (< 50 years old) *n* = 200; group 2 (≥ 50 years old) *n* = 161. *df* = degrees of freedom; CFI = Comparative Fit Index; RMSEA = Root Mean Score Error of Approximation; SRMR = Standardized Root Mean Residual. ^#^According to Cheung and Rensvold (2002) ΔCFI < 0.01 implies that the invariance assumption still holds.^1^

| Closeness of relationship: Model | χ²*(df)* | CFI | RMSEA  (90% CI) | SRMR | Model comp | Δχ²(Δ*df*) | ΔCFI | ΔRMSEA | Decision^#^ |
| --- | --- | --- | --- | --- | --- | --- | --- | --- | --- |
| M1 Configural invariance | 11.59(8) | .996 | .050  (.000-.108) | .01 | – | – | – |  | – |
| M2 Weak invariance (loadings) | 17.75(12) | .993 | .052  (.000-.099) | .03 | M1 | 6.17(4) | .003 |  | Accept |
| M3 Strong invariance (loadings  and intercepts) | 18.13(16) | .997 | .027  (.000-.076) | .03 | M2 | 0.37(4) | .004 |  | Accept |
| M4 Strict invariance (loadings, intercepts and residuals) | 23.22(21) | .997 | .024  (.000-.069) | .02 | M3 | 5.09(5) | .000 |  | Accept |

*Note.* *N* = 361; group 1 (spouse/first-degree relative) *n* = 318; group 2 (other) *n* = 43. *df* = degrees of freedom; CFI = Comparative Fit Index; RMSEA = Root Mean Score Error of Approximation; SRMR = Standardized Root Mean Residual. ^#^According to Cheung and Rensvold (2002) ΔCFI < 0.01 implies that the invariance assumption still holds.^1^
